# Feline blood donation: Description and adverse reactions from 29 201 donation events between 2019 and 2023

**DOI:** 10.1111/jvim.17215

**Published:** 2024-10-12

**Authors:** Samantha S. Taylor, Helena C.M. Ferreira, André F.P. Cambra, Giovanni Lo Iacono, Kamalan Jeevaratnam, Ignacio Mesa‐Sanchez, Rui R.F. Ferreira

**Affiliations:** ^1^ School of Veterinary Medicine University of Surrey Guildford United Kingdom; ^2^ Animal Blood Bank Porto Portugal; ^3^ Centro de Estudos de Ciência Animal (CECA) Universidade do Porto Porto Portugal

**Keywords:** anxiolysis, blood collection, blood donation, blood group, blood transfusion, cat, sedation

## Abstract

**Background:**

Feline blood transfusion is required for the treatment of various illnesses in cats, and the safety of donor cats is vital. Donor adverse reactions can include cardiorespiratory, venepuncture‐related, and behavioral abnormalities.

**Hypothesis/Objectives:**

To describe a large number of feline blood donation events and document use of sedation and anxiolysis, record volume of blood collected and describe the frequency, type, and risk factors for, adverse reactions.

**Animals:**

The study included 7812 individual cats and 29 201 donation events at a blood banking center over 5 years.

**Methods:**

Retrospective analysis of donation event records with signalment, donation volume, sedation status, donation number, and adverse reactions (acute and caregiver reported) recorded. Risk factors for adverse reactions were examined by stratifying data according to groups exposed to relevant predictors and calculating odds ratios with 95% and 99% confidence intervals (CIs).

**Results:**

Adverse reactions were uncommon (0.29%, 2.88/1000 donor events) and most commonly were cardiorespiratory (0.08%, 0.75/1000 donor events) or behavioral (0.06%, 0.62/1000 donor events). The only risk factor significantly associated with adverse reactions was conscious donation, with conscious donors 4.4 times more likely to have an adverse reaction (95% CI, 2.5‐7.9, *P* ≤ .0001).

**Conclusions and Clinical Importance:**

Feline blood donation is associated with a low rate of adverse reactions. Sedation should be considered to reduce adverse reactions, and the environment and interactions optimized to reduce donor stress. Caregiver education on care postdonation could reduce behavioral adverse reactions.

**Abbreviations**
FeLV: feline leukemia virus
FIV: feline immunodeficiency virus

## 1 INTRODUCTION

In the last decade, there has been a growth in the demand for feline blood and hence an increase in blood banking.^1^, ^2^, ^3^ To meet this growing need, developing and improving donor recruitment programs is essential and dependent on the willingness of caregivers to allow their pets to participate. Caregiver reservations about their cat donating,^4^ and ethical concerns around performing procedures on healthy animals,^5^ suggest donor safety continues to remain a concern. Adverse donor reactions should be minimized to increase the appeal to caregivers and maximize the wellbeing of donor cats.^3^, ^4^ There is extensive literature on the prevalence of postdonation reactions in human blood donors, with a complication rate of 6.3/1000 donations in a recent study.^6^ The most common reactions in humans are vasovagal, and can be influenced by stress and environmental conditions, with venepuncture‐related complications also reported.^6^, ^7^, ^8^ The frequency of adverse reactions after blood donation in cats seems to be low. Abreu et al,^9^ reported adverse reactions in 1.14% of 3690 donations from 1792 donors, the most frequent being behavioral changes (distress, lethargy, inappropriate urination) within the 5 days postdonation. Acute reactions in this study included weakness, pallor, tachypnea, and open‐mouth breathing in 0.22% of donors. Cardiorespiratory reactions were also reported in another study, at a frequency of 4.3% and only in the unsedated donor group.^10^ This study also showed increased donor anxiety and movement during donation in unsedated cats, although these were considered minor issues and the unsedated group did not have a higher frequency of unsuccessful donations or severe adverse reactions. The decision to sedate feline blood donors will be dictated by temperament as well as regional regulations.^11^ Factors affecting the frequency of adverse events in feline donors are understudied and hence the aim of the present study was to describe a large number of feline blood donation events, including the frequency and type of adverse reactions recorded, and examine potential factors associated with an increased risk of such reactions to improve the safety of the procedure for donor cats.

## 2 MATERIALS AND METHODS

### 2.1 Study design

In this retrospective study, records of feline blood donations at the Portuguese Animal Blood Bank between January 2019 and 2023 were examined. Each donation was described as a “donation event.” Part of these data included in the present study (3690 donation events) has been reported previously.^9^

### 2.2 Donation events

Donation events were included in the study if records for the donation were available for examination and included all of the following: unique donor identification number, donation date, donor body weight at donation event, volume of blood collected (total milliliter), use of sedation or anxiolysis, occurrence (including details) or nonoccurrence of donation adverse reactions. Additional information recorded if available included: blood type, breed, cat date of birth, sex, and predonation event hemoglobin. Donation volume in milliliters per kilogram and age were calculated.

### 2.3 Donor assessment

All donations were performed at the Portuguese Animal Blood Bank by trained veterinary personnel and with caregiver informed consent. Each donor was identified with a microchip to ensure donation traceability. Donors met the following criteria: no clinical signs of illness, receipt of medication or prior transfusion, calm temperament, weight >3.0 kg, between 1 and 10 years of age, current vaccination and flea and worm treatment, normal physical examination (if a heart murmur was present donor must have a normal echocardiogram). Additionally, for acceptance as a donor, predonation blood analysis required a hemoglobin ≥10 g/dL and a negative rapid test for feline immunodeficiency virus (FIV) antibody and feline leukemia virus (FeLV) antigen. Postdonation testing on each donation included PCRs for FeLV provirus, hemotrophic mycoplasmas, and *Bartonella henselae* and ELISA serology for FIV (antibody) and FeLV (antigen). Serum biochemistry and complete blood counts were performed on registered donor cats every 6 months at an external laboratory.

### 2.4 Donation process

After a period of acclimation in a quiet cat‐only area, the donor was weighed and examined, then wrapped in a blanket in sternal recumbancy, and a decision made to sedate or not to sedate (see below). An IV catheter was placed in the cephalic vein and if required, sedation was given IV. Blood was collected from the jugular vein (recording side used and alternating between donations) using a semiclosed system containing a syringe with anticoagulant (citrate, phosphate and dextrose) after clipping and aseptic preparation. As standard, a 10 mL/kg donation is collected, but subject to adjustment by the veterinary team according to donor evaluation and the donation event itself (eg, ease of donation collection, maximum guide 13 mL/kg). Upon needle removal, pressure was applied to the area and donors placed back in their carrier and monitored (respiratory rate and pattern, heart rate, mucous membrane color and demeanor) for at least 30 minutes or until fully recovered from sedation.

### 2.5 Sedation/anxiolysis

The decision to sedate or not to sedate a donor was based on observation of temperament and interpretation of current and prior body language and behavior at physical examination during current and prior donation events. Sedation protocol involved administration of a fixed quantity (0.15 mL total) of diazepam (0.54 mg), ketamine (2.71 mg) and butorphanol (0.14 mg) IV. Higher doses were administered, when necessary, based on donor anxiety and response to handling. Anxiolysis (gabapentin 100 mg/cat PO given 90 minutes before the donation appointment) was given if indicated by prior response to handling at donation events or if recommended by the cat's primary veterinarian.

### 2.6 Adverse reaction recording

Adverse reactions recorded in the donation event record were analyzed to calculate frequency of occurrence as a percentage of total donations and per 1000 donations. The following details were extracted from the records: clinical details of reactions occurring during and within 2 hours of the donation and information from a form (Appendix A) completed by a veterinary professional during a follow‐up phone call to the caregiver up to 5 days after the donation event. Treatment given and outcome, if available, was also recorded. Adverse reactions were classed as “acute” if they occurred during or within 2 hours of the blood collection and “caregiver reported” if they were reported at the follow‐up caregiver contact and recorded in the questionnaire.

Acute adverse effects were further categorized on review of the records by 1 author (S.S. Taylor) into “cardiorespiratory” if the cat showed signs of tachypnea, mucous membrane pallor, tachycardia, or suspected or confirmed hypotension (systolic blood pressure below 70 mmHg if measured), “venepuncture‐related” if hematoma or other abnormality at venepuncture site occurred or “other” to describe less frequent reaction types and details recorded.

Caregiver‐reported adverse effects in the 5 days between donation event and reporting were categorized as “lethargy” if the cat was reported to be quieter than normal, “sleepy,” or not moving as much, “behavioral” if the cat showed any abnormal behavior such as hissing at cohabitants, inappropriate urination, or change in behavior (other than lethargy), “venepuncture‐related” if the cephalic or jugular venepuncture sites showed any abnormalities, “gastrointestinal” if the cat showed vomiting, diarrhea, or inappetence, or “other” if outside those categories and details recorded.

If more than 1 type of adverse reaction occurred, all were recorded. If no treatment was recorded and the cat recovered spontaneously, this was recorded as “spontaneous recovery,” and if treatment was given, this was recorded as “treatment required” and details collated. Further outcome information was recorded if available including referral for further veterinary treatment or any further diagnostic or treatment data.

### 2.7 Statistical analysis

Data were recorded in Excel (Microsoft Excel for Microsoft 365 MSO Version 2212 Build 16.0.15928.20278). Descriptive statistics were used to describe the donor cat characteristics (age, sex, weight, breed) and volume of blood collected at donation events in milliliters per kilogram. Descriptive data were reported at median (range) for skewed variables and mean ± SD for normally distributed variables (Kolmogorov‐Smirnov test). To assess for nonlinear, monotonic correlations Spearman's rank correlation was used. Potential predictors (ie, the specific factors used to explain the observed outcome) and how they are associated with cats experiencing adverse reactions were also investigated. The following predictors were then assessed: total volume of blood collected at donation event (milliliters); volume blood collected per kilogram (milliliters per kilogram), weight, sedation status; blood type; age (days) at the time of donation, sex, hemoglobin concentration, breed. For this, the donor cat cohort was 1st divided into 2 groups: with and without adverse reactions. We then summarized the categorical predictors (sedation status; blood type, sex, breed) by the levels of the adverse reaction via a contingency table (adverse reaction YES/NO versus exposure to predictor YES/NO, see Table 3). This was followed by performing Pearson's Chi‐squared test for each single predictor (univariable analysis). Similarly, the continuous predictors (total volume of blood collected at donation event (milliliters); volume of blood collected per kilogram (milliliters per kilogram), weight, age (days) at the time of donation, and hemoglobin concentration) were summarized by evaluating mean, median, and quartiles for the groups developing and not developing an adverse reaction (Table 3). This was followed by performing a Kruskal‐Wallis rank sum test. The corresponding *P*‐value for each predictor was evaluated. The rationale for the univariable analysis was to eliminate variables that are unlikely to be associated with adverse reactions. This will render the subsequent multivariable analysis more efficient and transparent. If the *P*‐value was higher than the significance level of 0.25 (it is a common practice to choose a more conservative threshold for this preliminary screening),^12^ we considered the association between predictor and developing adverse reaction not significant, and the predictor was removed from the rest of the analysis. Importantly, significant predictors in the univariable analysis might no longer be significant in the multivariable analysis. The multivariable analysis was done by stratifying the data according to groups exposed to the relevant predictors. Odds ratios (ORs) for each predictor were provided with 95% and 99% confidence intervals (CIs) along with *P*‐values. For example, to investigate if weight was a risk factor, we divided the data into 2 groups (*strata*): the groups of cats whose weight was less than or equal to the median (4.3 kg; light donors) and the groups of cats whose weight was greater than the median (heavy donors; by using the median we ensured that both groups contained 50% of data). Then for each group we calculated the ratio of the odds of developing an adverse reaction in the sedated group and the odds of developing an adverse reaction in the unsedated group. If weight was not a risk factor, then the OR for the 2 groups was not expected to be significantly different.

It was anticipated that the donor's weight could be an effect modifier. To investigate this further we (i) estimated the proportion of cats developing an adverse reaction in 4 weight groups divided by quartile (rather than 2); (ii) stratified the donor cat cohort by sedation status and looked at the odds of developing an adverse reaction in the light and heavy cats and estimated their OR. Logistic regression was not used since the underlying assumptions were not satisfied, namely some of the observations come from repeated measurements of the same individual (the same donor can donate multiple times), and some predictors were highly correlated. This was corroborated by unsatisfactory diagnostics to check the validity of the assumptions in explorative regression analysis.

Finally, we explored the effect of the number of donations per cat in the sedated and unsedated groups on adverse effects. We calculated the number of cats with an adverse effect which subsequently had another donation event (ie, the adverse event did not prevent further donation). Then we divided the donor cat cohort into subgroups according to the number of blood donations and calculated the proportion of cats developing an adverse reaction within the subgroup (ie, the number of cats with an adverse reaction divided by the number of cats in the subgroup). Groups were then compared with a Chi‐squared test of independence assuming the groups were independent. Data analysis was performed using R.^13^

## 3 RESULTS

### 3.1 Donation events

**FIGURE 1:**
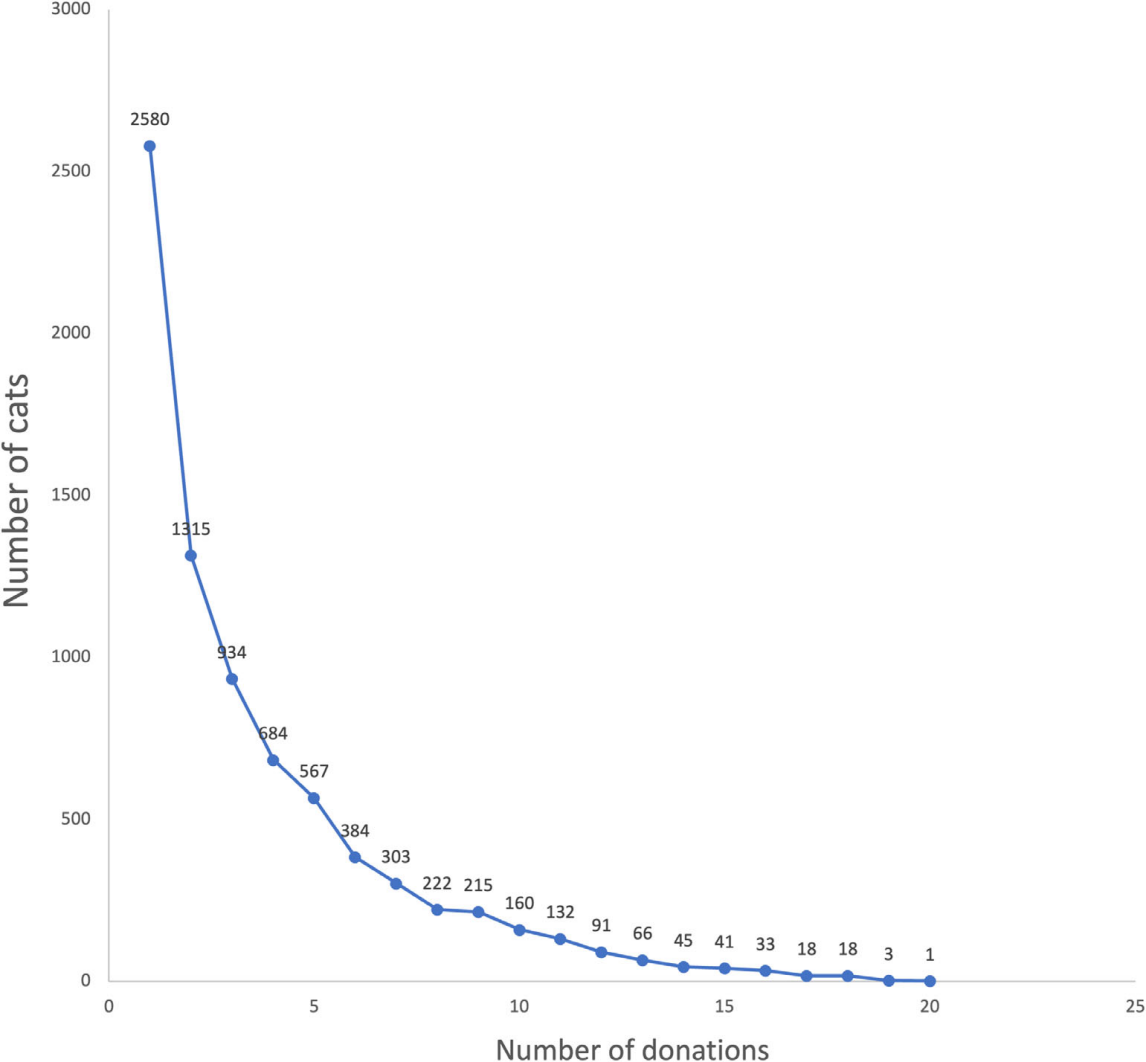
Chart illustrating the frequency of donation events per cat with most cats donating once.

A total of 29 201 donation event records from 7812 donors met inclusion criteria with each cat donating a median of 3 times (range, 1‐20). The frequency of donation is illustrated in Figure 1 with most cats donating once 2580/29 201 (8.8%).

#### 3.1.1 Donor cats

The donor cohort of 7812 individual cats included 7490/7812 (95.9%) type A, 231/7812 (3.0%) type B, 36/7812 (0.5%) type AB, and the remainder recorded as “not recorded” or “undifferentiated” (55/7812; 0.7%). There were more female (4182/7812; 53.5%) than male (3629/7812; 46.5%) donors and for those with breed recorded (2875 cats) most were domestic shorthairs (1923/2875; 66.9%), followed by Norwegian Forest cats (216/2875; 7.5%), Persian (137/2875; 4.8%), Maine Coon (133/2875; 4.6%), British shorthair (107/2875; 3.7%), and 13 pure breeds with less than 100 cats included. The median age at donation was 50 months (range, 12‐131) and median body weight 4.3 kg (range, 3.0‐12.9 kg). Median hemoglobin at donation event was 14 g/dL (range, 10.0‐24.6).

#### 3.1.2 Donation volume in milliliters per kilogram

**FIGURE 2:**
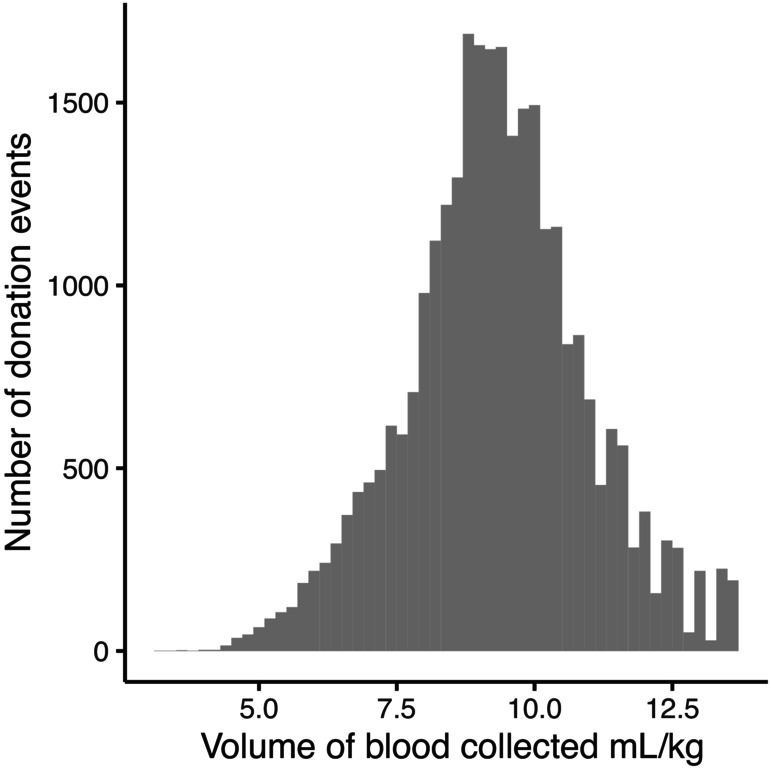
Chart illustrating volume of blood collected per kilogram at each donation event.

**FIGURE 3:**
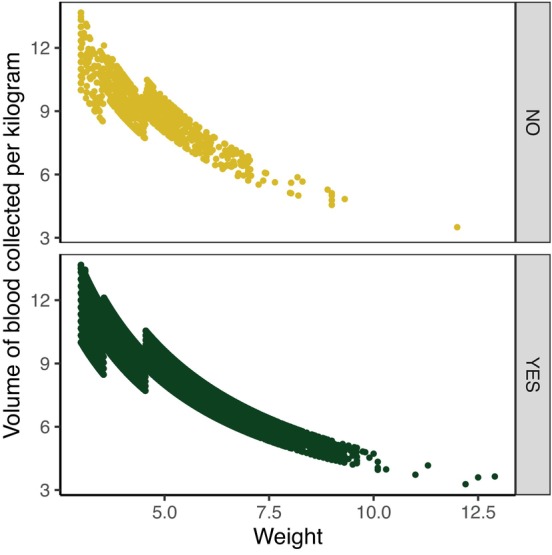
Scatterplot showing donation volume in milliliters per kilogram, illustrating correlation between lower body weight and higher donation volume (milliliters per kilogram) stratified by sedation status. Spearman's rank correlation in the sedated group: ρ = −0.8732 (*S* = 6.7983e+12, *P*‐value <2.2e‐16); Spearman's rank correlation in the unsedated group: ρ = −0.8741 (*S* = 648 911 968, *P*‐value <2.2e‐16).

The median volume of blood collected at donation events was 9.3 mL/kg (range, 3.3‐13.7; Figure 2). It is worth noting that the volume of blood collected in milliliters per kilogram strongly correlated with the weight of the cats, as shown in Figure 3, with larger volume of blood per kilogram collected from lower weight cats.

#### 3.1.3 Sedation/anxiolysis

Of the 29 201 donation events, most involved donor sedation (27 925/29 201; 95.6%) with only 4.4% (1276/29 201) unsedated. A total of 7618/27 925 (27.3%) donation events included cats given more than 0.15 mL total dose of the sedation protocol. Oral gabapentin had been given to donors before 54/29 201 (0.18%) donation events, but additional IV sedation was required in all but 1 of these donation events.

#### 3.1.4 Adverse reactions

**FIGURE 4:**
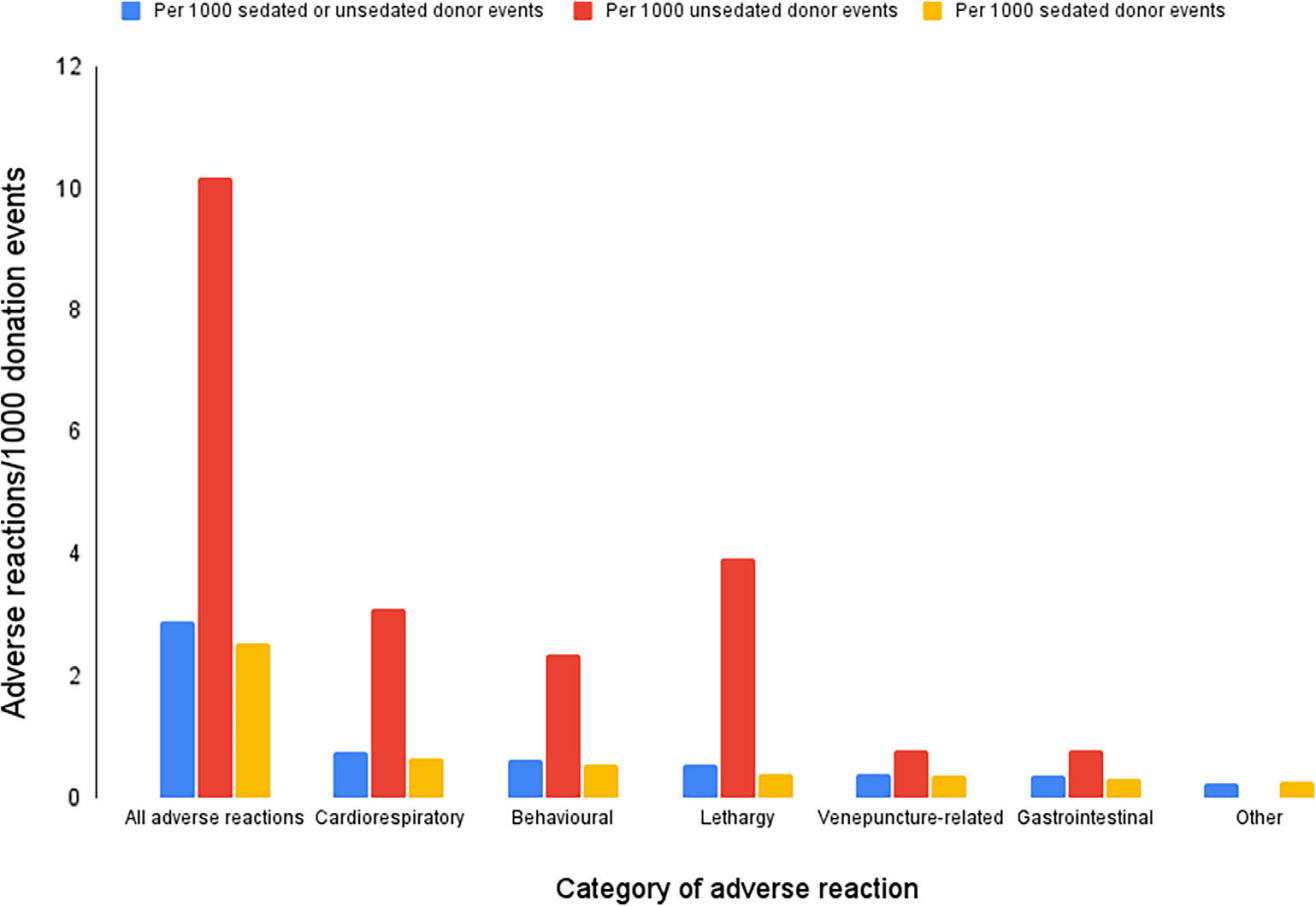
Bar chart showing adverse reactions per 1000 sedated or unsedated donor events.

Adverse reactions are described in Table 1 and Figure 4 and occurred after 84/29 201 (0.29%) donation events in 84 individual cats equating to 2.88/1000 donation events. Most adverse events were caregiver‐reported (57/29 201, 0.20%) with fewer acute adverse reactions (27/29 201, 0.09%). Adverse reactions were reported in 70/27 925 (0.25%) of sedated donor events and 14/1276 (1.1%) unsedated donor events.

**TABLE 1:** Adverse reactions associated with 29 201 donation events.

		Number of donation events where adverse reaction occurred/total donation events (%)			Number of adverse reactions per 1000 donor events		
Category of adverse reaction		All donation events (29 201)	Adverse reactions/sedated donation events (% of 27 925)	Adverse reactions/unsedated donation events (% of 1276)	All donation events	Per 1000 sedated donation events	Per 1000 unsedated donation events
All categories		84/29 201 (0.29)	70/27 925 (0.25)	14/1276 (1.09)	2.88	2.51	10.97
Acute 27/29 201 (0.09)	Cardiorespiratory	22/29 201 (0.08)	18/27 925 (0.07)	4/1276 (0.31)	0.75	0.65	3.14
	Venepuncture‐related	1/29 201 (0.00)	0/27 925 (0.00%)	1/1276 (0.08)	0.03	0.00	0.78
	Other	4/29 201 (0.01)	4/27 925 (0.01)	0/1276 (0.00)	0.14	0.14	0.00
Caregiver reported 57/29 201 (0.20)	Behavioral	18/29 201 (0.06)	15/27 925 (0.05)	3/1276 (0.24)	0.62	0.54	2.35
	Lethargy	16/29 201 (0.06)	11/27 925 (0.04)	5/1276 (0.39)	0.55	0.39	3.92
	Venepuncture‐related	10/29 201 (0.03)	10/27 925 (0.04)	0/1276 (0.00)	0.35	0.36	0.00
	Gastrointestinal	10/29 201 (0.03)	9/27 925 (0.03)	1/1276 (0.08)	0.36	0.32	0.78
	Other	3/29 201 (0.01)	3/27 925 (0.01)	0/1276 (0.00)	0.11	0.11	0.00

##### Acute adverse reactions

The most frequent acute adverse reaction was cardiorespiratory in 22 cats (22/29 201, 0.08%). Most affected cats were described as “hypotensive” or suffering a “vagal reaction” (18/22 cats) and 4 cats were described as tachypneic (4/22). Of the 22 cats, most, (18/22), were classified as “treatment required” including fluid therapy post donation (13/18), butorphanol and oxygen (2/18), referral to the primary veterinarian (2/18), and oxygen and feeding alone (1/18). All but the 2 cats referred to the primary veterinarian were discharged without further treatment. Four cats were classed as having a spontaneous recovery and were discharged.

Of the 2 cats referred to the primary veterinarian; both were later found to be hyperbilirubinemic (not clinically evident at donation event) with 1 diagnosed with biliary obstruction and the other without a definitive diagnosis. Further acute adverse reactions (5/27) included 1 venepuncture‐related (hematoma at jugular site), and 4 classed as “other” including 2 cats noted to be icteric after donation and referred to the primary veterinarian (no further information recorded), 1 with sudden hyperextension of limbs that resolved spontaneously, and 1 described as “over‐sedated” that also recovered spontaneously.

##### Caregiver‐reported adverse reactions

The most frequent caregiver‐reported adverse reaction was behavioral (18/29 201; 0.06%) and occurred within 24 h of donation, with further details in Table 2. All cats had a spontaneous recovery. Lethargy was reported by caregivers of 16 cats and for 9/16 duration was recorded between 24 and 72 hours before spontaneous recovery. Ten cats (10/29 201, 0.03%,) were reported to have venepuncture‐related adverse reactions including 7/10 with a rash at clipping site (jugular vs cephalic not recorded), 2/10 with a hematoma (1 jugular, 1 cephalic), and 1 cat with edema and hematoma at the cephalic IV catheter site that required assessment by the primary veterinarian. Ten caregivers reported gastrointestinal adverse reactions including 6/10 cats which vomited and 4/10 with reduced appetite, 2 of which were later diagnosed with underlying pathology by the primary veterinarian (1 pancreatitis, 1 lingual ulcer). Other caregiver‐reported adverse reactions (in 3 cats) included 1 cat with episodes of pyrexia and 1 with unilateral forelimb lameness (same limb as IV catheter had been placed) both with spontaneous resolution and 1 with hematuria referred to the primary veterinarian (no further information documented).

**TABLE 2:** Behavioral adverse reactions reported by caregivers of cats following 29 201 donor events.

Behavioral adverse reaction reported by caregiver	Total number of behavioral adverse reactions (% of total behavioral adverse reactions)	Number of behavioral adverse reactions where cat was sedated (% of total number of behavioral adverse reactions)	Number of behavioral adverse reactions where cat was unsedated (% of total number of behavioral adverse reactions)
All behavioral adverse reactions	18	15 (83.3)	3 (16.7)
Aggression to caregiver or other animals in the home	9 (50.0)	7 (38.9)	2 (11.1)
Inappropriate urination	3 (16.7)	3 (16.7)	0 (0.0)
Increased vocalization	3 (16.7)	3 (16.7)	0 (0.0)
Change in behavior/strange behavior	3 (16.7)	2 (11.1)	1 (5.6)

Only 3 cats were recorded as having more than 1 adverse reaction, 1 caregiver‐reported with lethargy and behavioral (aggression), 1 with lethargy and gastrointestinal (inappetence), and 1 cat with an acute reaction; cardiorespiratory (hypotensive) and other (icteric; described above).

#### 3.1.5 Factors associated with adverse reactions

Table 3 shows summary statistics, along with statistical tests, which compared the distributions of a set of independent predictors stratified by the adverse reaction status, with the following findings:

**TABLE 3:** Summary statistics for a set of predictors stratified by the adverse reaction status.

	Adverse reaction		*P*‐value
	No	Yes	
Sedation status			<.001^a^
No	1262 (4.3%)	14 (16.7%)	
Yes	27 855 (95.7%)	70 (83.3%)	
Weight (kg)			.007^b^
Count	29 117	84	
Median	4.30	4.00	
Mean	4.52	4.31	
Q1,Q3	3.70, 5.10	3.32, 4.82	
Blood type			.896^a^
A	27 831 (95.6%)	80 (95.2%)	
AB	90 (0.3%)	0 (0.0%)	
B	1134 (3.9%)	4 (4.8%)	
Undifferentiated	62 (0.2%)	0 (0.0%)	
Volume blood collected (mL)			.041^b^
Count	29 117	84	
Median	41.00	40.00	
Mean	40.42	39.631	
Q1,Q3	37.00, 43.000	36.75, 42.25	
Volume of donation per kilogram (mL/kg)			.009^b^
Count	29 117	84	
Median	9.28	9.65	
Mean	9.31	9.74	
Q1,Q3	8.32, 10.29	8.75, 11.01	
Age (days) at donation			.536^b^
Count	22 325	70	
Median	1518.00	1538.50	
Mean	1671.63	1743.43	
Q1,Q3	955.00, 2293.00	955.00, 2350.25	
Breed			.337^a^
Not recorded	402 (1.4%)	0 (0.0%)	
American curl	9 (0.0%)	0 (0.0%)	
Bengal	29 (0.1%)	0 (0.0%)	
Bobtail	2 (0.0%)	0 (0.0%)	
British longhair	2 (0.0%)	0 (0.0%)	
British short hair	357 (1.2%)	2 (2.4%)	
Domestic shorthair	11 388 (39.1%)	48 (57.1%)	
Undetermined	14 230 (48.9%)	26 (31.0%)	
Maine Coon	376 (1.3%)	0 (0.0%)	
Munchkin	4 (0.0%)	0 (0.0%)	
Norwegian Forest cat	1140 (3.9%)	4 (4.8%)	
Persian	556 (1.9%)	1 (1.2%)	
Ragdoll	67 (0.2%)	0 (0.0%)	
Scottish fold	46 (0.2%)	0 (0.0%)	
Scottish straight	66 (0.2%)	1 (1.2%)	
Siamese	358 (1.2%)	2 (2.4%)	
Siberian	19 (0.1%)	0 (0.0%)	
Somali	4 (0.0%)	0 (0.0%)	
Sphynx	42 (0.1%)	0 (0.0%)	
Toyger	20 (0.1%)	0 (0.0%)	
Sex			.393^a^
Female	15 509 (53.3%)	51 (60.7%)	
Male	13 607 (46.7%)	33 (39.3%)	
Hemoglobin (g/dL)			.059^b^
Count	28 498	78	
Median	14.00	13.650	
Mean	13.99	13.65	
Q1,Q3	12.80, 15.10	12.63, 14.38	

*Note*: For simplicity, each donation is treated as a separated event (even if from the same donor).

^a^ Pearson's Chi‐squared test.

^b^ Kruskal‐Wallis rank sum test.

The univariable analysis clearly indicated that sedation status was strongly associated with the occurrences of adverse reactions. Age at donation, breed, sex and blood type, were not associated with increased frequency of adverse reaction (*P*‐value >.25) and were removed from subsequent multivariable analysis. The findings were less clear with the following, potentially correlated, predictors: weight, total volume of blood collected, and volume of blood collected (milliliters per kilogram), and hemoglobin.

Total volume of blood collected and volume of blood collected per kilogram (milliliters per kilogram), were highly correlated, as expected since the volume of blood collected (milliliters per kilogram) is calculated as the total volume of blood collected divided by the cat's weight. More interesting is the correlation between volume of blood collected per kilogram (milliliters per kilogram) and weight. Figure 3 shows that the volume of blood per kilogram decreases with the weight of cat (thus lighter cats donate higher volume of blood relative to their weight), making 1 of the factors a potential confounder. For completeness results are illustrated stratified by sedation status. Data were therefore stratified by sedation status predictor with highest significance in the univariable analysis for adverse reaction as shown in Table 3 and weight by diving donors in 2 groups using 4.3 kg (the median) as threshold. The Kruskal‐Wallis rank sum test for each individual variable in the stratified groups confirmed that total volume of blood collected, and volume of blood collected (milliliters per kilogram), and hemoglobin (as well as age, breed and sex) were not associated with adverse reactions (all *P*‐values for each predictor range from 0.137 to 0.885) as shown in Table 4. Thus, the predictors potentially associated with adverse reaction were sedation status and weight.

**TABLE 4:** Summary statistics for a set of predictors stratified by the adverse reaction status and after stratifying for weight.

Weight ≤ median (4.3 kg).			
	Adverse reaction		*P*‐value
	No	Yes	
Volume blood collected (mL)			.641
Count	14 740	54	
Median	38.00	38.00	
Mean	37.65	37.56	
Q1,Q3	35.00, 40.00	35.00, 40.00	
Volume of donation per kilogram (mL/kg)			.138
Count	29 117	84	
Median	9.28	9.65	
Mean	9.31	9.74	
Q1,Q3	8.32, 10.29	8.75, 11.01	
Hemoglobin (g/dL)			.288
Count	28 498	78	
Median	14.00	13.65	
Mean	13.99	13.65	
Q1,Q3	12.80, 15.10	12.63, 14.38	
Volume blood collected (mL)			.885
Count	14 377	30	
Median	43.00	43.50	
Mean	43.25	43.37	
Q1,Q3	14 377	30	
Volume of donation per kilogram (mL/kg)			.832
Count	14 377	30	
Median	8.32	8.33	
Mean	8.16	7.97	
Q1,Q3	7.45, 9.03	6.854, 9.37	
Hemoglobin (g/dL)			.137
Count	14 127	29	
Median	14.10	13.70	
Mean	14.12	13.65	
Q1,Q3	13.00, 15.30	13.00, 14.40	

*Note*: Since all predictors are continuous, Kruskal‐Wallis rank sum test was used.

Table 5 shows the crude OR for sedation status, as well as stratified by weight and the ORs for the factor weight in the sedated and unsedated group. The analysis shows lack of sedation was a risk factor for adverse reactions with an OR being 4.4 times than in the sedated group (95% CI, 2.5‐7.9). The findings were qualitatively similar for the light and heavy donors, however, the relative difference in the magnitudes of the ORs in the 2 groups was about 23%; furthermore, in contrast with the light group, the 99% CI for the OR in the heavy donors spans over 1, although the association is still significant according to predetermined criterion for significance level.

**TABLE 5:** Measure of the risk of developing an adverse reaction for sedation status (ie, ratio of the odds of developing an adverse reaction in the sedated group and the odds of developing an adverse reaction in the unsedated group), stratified by weight and measure of the risk of developing an adverse reaction for the factor “weight” (ie, the odds of developing an adverse reaction in lighter group divided by the odds of developing an adverse reaction in heavier group) in the sedated and unsedated groups.

Factor	OR	(95% CI) and (99% CI)	*P*‐value (Fisher)
Sedation status for all donors	4.4	(2.5‐7.9) (2.1‐9.4)	1.56E‐5
Sedation status in donors ≤4.3 kg	4.7	(2.4‐9.4) (1.9‐11.7)	.00016
Sedation status in donors >4.3 kg	3.63	(1.3–10.4) (0.905‐14.5)	.032
Weight less than 4.3 kg in sedated donor group (ie, the odds of developing an adverse reaction in lighter group divided by the odds of developing an adverse reaction in heavier group)	1.66	(1.0‐2.7) (0.88‐3.1)	.042
Weight less than 4.3 kg in unsedated donor group (ie, the odds of developing an adverse reaction in lighter group divided by the odds of developing an adverse reaction in heavier group)	2.5	(0.8‐8.1) (0.55‐11.7)	.17

Abbreviations: CI, confidence interval; OR, odds ratio.

(*P*‐value = .032 < .05, and 95% CI, 1.3‐10.4). This suggested that donor's weight could be an effect modifier. To investigate we

**FIGURE 5:**
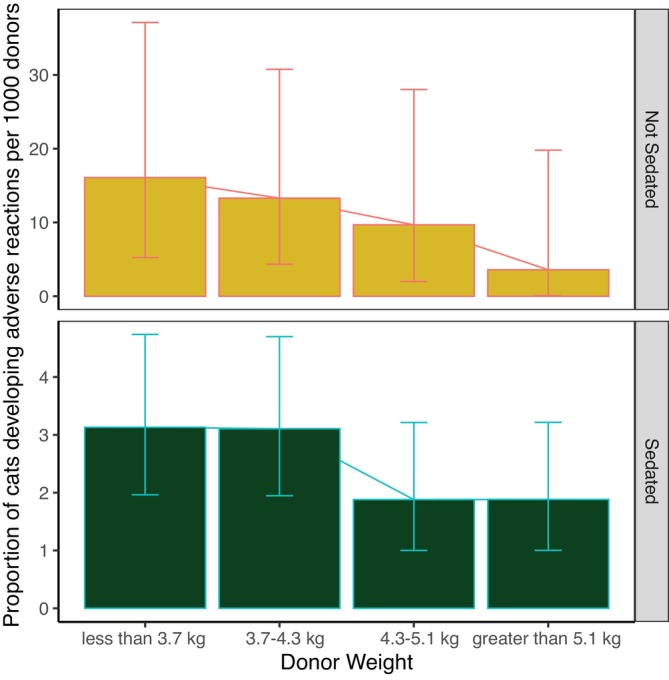
Proportion of cats developing adverse reaction divided by weight groups for the sedated and unsedated cats. The error bars represent the 95% confidence interval (CI) using Clopper‐Pearson method. Scale on the y‐axis is different for visual purposes.

1. estimated the proportion of cats developing an adverse reaction in 4 weight groups divided by quartile, corresponding to the 4 intervals: <3.1 kg, between 3.1 kg and 4.3 kg, between 4.3 kg and 5.1 kg and >5.1 kg, (Figure 5); and
2. stratified the donor cat cohort by sedation status and looked at the odds of developing an adverse reaction in the light and heavy cats and estimated their OR (Table 5).

In the unsedated group, the association between weighing less than 4.3 kg and developing an adverse reaction was not significant (*P*‐value = .17 > .05) and further supported by both CIs of the OR containing 1. In the sedated group, based on the predetermined criterion for significance level, weighing less than 4.3 kg was a risk factor for developing an adverse reaction. Although the 95% CI suggested that weighing less than 4.3 kg was a risk factor for developing an adverse reaction, the much more stringent 99% CI did not.^14^ Figure 5 shows that the proportion of cats developing an adverse reaction decreases according to the weight of donors in both sedated and unsedated groups; however, the chi‐squared test of independence showed that association between weight groups and the proportion of cats developing an adverse reaction was not significant (χ^2^ = 4.2755, *P*‐value = .25 for sedated cats; χ^2^ = 2.3856, *P*‐value = .52 in unsedated cats). Taken together, there is not compelling evidence that weight is a risk factor for developing adverse reactions in the sedated or unsedated donor groups.

Most cats (73/84; 86.9%) with an adverse reaction had a subsequent donation event (ie, the cat donated again) and there was no significant association between developing an adverse reaction and the number of donations (χ^2^ = 17.25, *P*‐value = .49 for sedated cats; χ^2^ = 18.47, *P*‐value = .20 in unsedated cats).

## 4 DISCUSSION

Our study includes the largest number of feline donation events reported to date and shows a low number of adverse events. A total of 29 201 donation events from 7812 donors were included and adverse events recorded after 84 donation events (0.29%; 2.88/1000 donation events). The most frequent adverse events were caregiver‐reported (57/84 adverse events), mainly behavioral (18/57) and lethargy (16/57). Acute adverse reactions (27/84) were mainly cardiorespiratory (22/27). The only parameter significantly increasing the risk of adverse reaction was unsedated (conscious) donation. The data imply that being a lighter cat (<4.3 kg) could be a risk factor if sedated for donation, but the findings are not conclusive. Similarly, the proportion of cats developing an adverse reaction increases as the donor body weight decreases, but the association was not significant. Further research is needed to investigate if donor body weight is an effect modification.

Previous studies of donor adverse reactions with smaller numbers of cats have shown variable levels of adverse reactions (described also as unexpected events) between 1.14%,^9^ and 46.96%,^10^ but Doolin et al,^10^ included anxiety at donation and inadequate donation volume as unexpected events, not factors included as adverse reactions in this study because of lack of specific recording and variable definitions of “anxiety” and “inadequate donation volume.” The adverse reaction rate reported in the present study is lower than the overall complication rate in human donors of 6.3/1000 donations reported in a large study by the International Haemovigilance Network.^6^

Unsedated/conscious donors in the current study were 4.4 times more likely to have an adverse reaction. Conscious donation has appeal because of perceived risks of sedation, additional interventions and time for recovery, but even in a cat friendly environment, is likely to increase donor stress from restraint and potential pain from venipuncture, as well as increasing the risk of movement and inadequate donation volumes, although the latter was not more likely in unsedated donors in 1 study.^10^ The adequacy of donation volume was not specifically analyzed in the present study. The higher proportion of owners of unsedated/conscious donors reporting behavioral adverse effects could also suggest ongoing postdonation negative impacts on emotional health/mental wellbeing from conscious donation.

The effect of sedation and anesthesia on feline blood donors has been studied,^10^, ^15^, ^16^ with cardiovascular effects of various drugs on blood donors reported. The combination of sedative agents used in the studied donor program (diazepam, ketamine, butorphanol) has not been specifically examined. Given the low rate of adverse reactions from a very large number of sedated cats reported here, this protocol could be considered for other programs. Given the aim of maximizing the physical and mental wellbeing of donor cats, the findings of the present study support a recommendation to sedate feline blood donors to potentially reduce adverse reactions even further.

The use of gabapentin has not been studied in feline donor programs but has been shown to reduce anxiety associated with veterinary visits,^17^ with a lack of cardiovascular effects in healthy adult cats,^18^ making predonation appointment anxiolysis potentially beneficial. In the current study only 54 cats received gabapentin, and all but 1 required further sedation. However, advice to give gabapentin resulted from reports of the cat's demeanor possibly selecting for cats requiring sedation to donate, plus larger cats could require more than 100 mg, hence conclusions cannot be drawn on the benefits of gabapentin for anxiolysis from our study.

In human medicine, vasovagal reactions are the most common acute complication related to blood donation^19^ and reduce the likelihood of repeat donations. In the present study, perhaps surprisingly, most cats with an adverse reaction did donate again. Multiple factors contribute to vasovagal reactions in humans such as direct effects of blood removal, stress and relative hypovolemic states.^20^ Doolin et al,^10^ noted a small number of cats (4.3%) with cardiovascular or respiratory distress (hence not all vasovagal), but this figure is higher than the 0.08% in the present study. Given the impact of stress and a noisy environment on vasovagal reactions in human donors,^20^ the low levels of cardiorespiratory (and likely vasovagal) reactions in feline donors in our study could relate to the extensive experience and training of the veterinary staff and following of Cat Friendly Clinic principles,^21^ in a cat friendly environment,^22^ reducing donor stress, as well as the use of sedation in the majority of donors. Donor programs should prioritize stress reduction in the peri‐donation period to reduce adverse reactions.

In the described donor program, fluid therapy is not routinely administered. Various fluid therapy protocols are described for donor cats,^23^, ^24^ including arbitrary advice on providing fluids if 20% of blood volume is collected.^24^ However, a previous study of 3690 donations made without fluid therapy,^9^ reported only a low level of adverse reactions. Additionally, a recent study of 100 feline donors showed no significant difference in systolic blood pressure (or rate of owner observed changes postdonation) between cats receiving IV fluid therapy postdonation and those that did not.^25^ With data from the current study showing very infrequent cardiorespiratory adverse effects, the potential benefits of fluid therapy should be weighed against the time, costs and additional intervention and stress of fluid therapy. However, close monitoring and prompt intervention is clearly indicated should cats show acute adverse reactions such as hypotension. Prompt identification and response, plus the experience of the veterinary team involved in the described donor program likely contributed to the rapid recovery of most affected cats in our study.

Following statistical analysis, volume of blood collected per kilogram was not significantly associated with adverse reactions. Recommendations of 10 to 12 mL/kg or 40 to 50 mL per cat are often provided,^3^, ^11^, ^24^ and in the current study, the median volume collected was 9.3 mL/kg. Given the lack of association with, and low rate of, adverse reactions, donation volumes of 10 mL/kg can be considered with confidence.

Weight limits for donor programs vary according to official regulations. In the described donor program, there is a lower weight limit of 3.0 kg and lighter cats had a higher milliliters per kilogram donation volume and possibly higher risk of adverse reactions. In humans, lower body mass index has been associated with increased postdonation reactions,^26^ and could be predictive of vasovagal reactions.^27^ Given the higher milliliters per kilogram donations from smaller cats documented in the present study, and potential association with more frequent adverse reactions; feline donor programs should consider limiting donation volumes to 10 mL/kg for smaller cats, or restricting donors to cats over 3.5 kg for example, and the blood bank described here has updated protocols accordingly.

Behavioral adverse reactions were the most common caregiver‐reported reactions with the most frequent being aggression to other animals or to caregivers. Nonrecognition or redirected aggression can occur when cats are reintroduced to other animals, or people in the home after a period away and is generally not well studied.^28^ It might occur because of loss of group scent and relate to fear and frustration emotions of the donor cat.^21^ The occurrence of these reactions in the present study was low, but could potentially be further reduced by measures to initially separate donor cats in a different room to other pets and people, encourage mingling of scents from the home and allow the level of arousal of the returning cat to reduce before reintroduction. Donor caregiver education and communication is vital as such adverse reactions could easily deter future donations.

Limitations of the current study include the retrospective nature including reliance on accurate recording of adverse reactions in the records. Caregiver‐reported adverse reactions could also be unreliable and do not include observation by a veterinary professional. Blood pressure was not routinely measured after transfusion or in cats showing cardiorespiratory adverse reactions, so descriptions of hypotension are likely assumed. Predonation echocardiography was performed in cats with heart murmurs, but the presence or absence of murmurs is an insensitive indicator of underlying cardiac disease.^29^ Additionally, the decision to sedate cats for donation is made according to the cat's response to handling. This could select for cats with certain temperaments being included in sedated or not sedated groups (eg, more tolerant cats not being sedated) and affect results. The program uses a standard volume of sedation, adjusted according to reaction to handling, which could have affected results. The current study includes data from a large and experienced donor program with extensive staff training in donation collection and cat friendly interactions likely contributing to the low adverse reaction figures, which might not be extrapolatable to other donor programs or emergency collection of blood in veterinary clinics by less experienced staff and donors with less robust assessment and inclusion criteria. In the current study, despite the data suggesting lighter sedated cats might have an increased risk of adverse reactions, evidence was not adequate to reach this conclusion. The association of weight and adverse reactions could be further researched by selecting the relevant variables using a strategy such as Purposeful Selection of Variables,^12^ which includes variables using a systematic and interactive approach based on their statistical significance, and minimizing potential confounding effects.

In conclusion, feline blood donation involving a trained and experienced veterinary team is associated with a low rate of complications. The most frequent adverse reactions are behavioral and cardiorespiratory and most spontaneously resolve. Adverse reactions were more likely in conscious donors and hence sedation should be considered. Additionally, advising caregivers of donors on correct reintroduction to the household, and limiting donation volumes to 10 mL/kg for smaller cats, is recommended to maximize donor physical and mental wellbeing.

## CONFLICT OF INTEREST DECLARATION

Authors declare no conflict of interest.

## OFF‐LABEL ANTIMICROBIAL DECLARATION

Authors declare no off‐label use of antimicrobials.

## INSTITUTIONAL ANIMAL CARE AND USE COMMITTEE (IACUC) OR OTHER APPROVAL DECLARATION

Authors declare no IACUC or other approval was needed.

## HUMAN ETHICS APPROVAL DECLARATION

Authors declare human ethics approval was not needed for this study.

## APPENDIX A POST DONATION ADVERSE REACTIONS—PHONE CALL PROTOCOL

### A.1

**Table:**

The phone calls will be performed up to 5 days after donation. The owners will be informed on the day of donation that this phone call will be performed to evaluate possible reactions and to help with our ongoing study. Question owner to ensure everything is ok with the donor or if any suspected adverse events occurred, and only then we should discuss more specifics related with the described issue. A questionnaire should be completed for each donor, and should include the following questions:
**Has your pet suffered any of the following?** (*when did it start?/for how long?/required medical assistance?*):
a. *Cutaneous rash*
i. *Neck*
ii. *Forelimb*
b. *Hematoma*
i. *Neck*
ii. *Forelimb*
c. *Lack of appetite*
d. *Bleeding*
i. *Neck*
ii. *Forelimb*
e. *Increased respiratory rate*
f. *Pale mucous membranes (gums)*
g. *Lethargy*
h. *Abnormal behavior*
i. *Fainting*
**Any additional comments?**
