# Erratum for “Feline Blood Donation: Description and Adverse Reactions From 29 201 Donation Events Between 2019 and 2023”

**DOI:** 10.1111/jvim.70194

**Published:** 2025-07-25

S. S. Taylor, H. C. M. Ferreira, A. F.P. Cambra, G. L. Lacono, K. Jeevaratnam, I. Mesa‐Sanchez, and R. R. F. Ferreira, “Feline Blood Donation: Description and Adverse Reactions From 29 201 Donation Events Between 2019 and 2023,” *Journal of Veterinary Internal Medicine* 38, no. 6 (2024), https://doi.org/10.1111/jvim.17215.

In the above‐mentioned article, Table 4 “Summary statistics for a set of predictors stratified by the adverse reaction status and after stratifying for weight” had some entries from Table 3 “Summary statistics for a set of predictors stratified by the adverse reaction status” mistakenly mixed in and a missing subheader of “Weight > median (4.3 kg).” The corrected entries are highlighted in red bold fonts. The correct table is displayed here.

**TABLE 4:** Summary statistics for a set of predictors stratified by the adverse reaction status and after stratifying for weight.

	Adverse reaction		*p‐value*
	No	Yes	
Weight ≤ median (4.3 kg)			
Volume blood collected (mL)			0.641
Count	14740	54	
Median	38.00	38.00	
Mean	37.65	37.56	
Q1, Q3	35.00, 40.00	35.00, 40.00	
Volume of donation per kilogram (mL/kg)			0.138
Count	**14740**	**54**	
Median	**10.26**	**10.53**	
Mean	**10.42**	**10.72**	
Q1, Q3	**9.52, 11.11**	**9.60, 11.53**	
Hemoglobin (g/dL)			0.288
Count	**14371**	**49**	
Median	**13.90**	**13.60**	
Mean	**13.85**	**13.65**	
Q1, Q3	**12.60, 15.00**	**12.60, 14.20**	
**Weight > median (4.3 kg**)			
Volume blood collected (mL)			0.885
Count	14377	30	
Median	43.00	43.50	
Mean	43.25	43.37	
Q1, Q3	**41.00, 46.00**	**41.00, 46.00**	
Volume of donation per kilogram (mL/kg)			0.832
Count	14377	30	
Median	8.32	8.33	
Mean	8.16	7.97	
Q1, Q3	7.45, 9.03	6.85, 9.37	
Hemoglobin (g/dL)			0.137
Count	14127	29	
Median	14.10	13.70	
Mean	14.12	13.65	
Q1, Q3	13.00, 15.30	13.00, 14.40	

*Note*: Since all predictors are continuous, the Kruskal‐Wallis rank sum test was used.

We apologize for this error.

